# Exogenous monocyte myeloid-derived suppressor cells ameliorate immune imbalance, neuroinflammation and cognitive impairment in 5xFAD mice infected with *Porphyromonas gingivalis*

**DOI:** 10.1186/s12974-023-02743-8

**Published:** 2023-03-02

**Authors:** Xiao Cheng, Li Chi, Tianqiong Lin, Fengyin Liang, Zhong Pei, Jianbo Sun, Wei Teng

**Affiliations:** 1 Hospital of Stomatology, Guangdong Provincial Key Laboratory of Stomatology, Institute of Stomatology, Guanghua School of Stomatology, Sun Yat-Sen University, Guangzhou, China; 2 Dongguan Key Laboratory of Chronic Inflammatory Diseases, The First Dongguan Affiliated Hospital, Guangdong Medical University, Dongguan, China; 3 Department of Neurology, The First Affiliated Hospital, Sun Yat-Sen University; Guangdong Provincial Key Laboratory of Diagnosis and Treatment of Major Neurological Diseases; National Key Clinical Department and Key Discipline of Neurology, No.58 Zhongshan Road2, Guangzhou, 510080 China

**Keywords:** *Porphyromonas gingivalis*, Alzheimer’s disease, mMDSCs, Cognitive impairment

## Abstract

**Background:**

Periodontitis is closely associated with the pathogenesis of Alzheimer’s disease (AD). *Porphyromonas gingivalis* (Pg), the keystone periodontal pathogen, has been reported in our recent study to cause immune-overreaction and induce cognitive impairment. Monocytic myeloid-derived suppressor cells (mMDSCs) possess potent immunosuppressive function. It is unclear whether mMDSCs-mediated immune homeostasis is impaired in AD patients with periodontitis, and whether exogenous mMDSCs could ameliorate immune-overreaction and cognitive impairment induced by Pg.

**Methods:**

To explore the influence of Pg on cognitive function, neuropathology and immune balance in vivo, 5xFAD mice were treated with live Pg by oral gavage, three times a week for 1 month. The cells of peripheral blood, spleen and bone marrow from 5xFAD mice were treated with Pg to detect the proportional and functional alterations of mMDSCs in vitro. Next, exogenous mMDSCs were sorted from wild-type healthy mice and intravenously injected into 5xFAD mice that were infected with Pg. We used behavioral tests, flow cytometry and immunofluorescent staining to evaluate whether exogenous mMDSCs could ameliorate the cognitive function, immune homeostasis and reduce neuropathology exacerbated by Pg infection.

**Results:**

Pg exacerbated cognitive impairment in 5xFAD mice, with the deposition of amyloid plaque and increased number of microglia in the hippocampus and cortex region. The proportion of mMDSCs decreased in Pg-treated mice. In addition, Pg reduced the proportion and the immunosuppressive function of mMDSCs in vitro. Supplement of exogenous mMDSCs improved the cognitive function, and enhanced the proportions of mMDSCs and IL-10^+^ T cells of 5xFAD mice infected with Pg. At the same time, supplement of exogenous mMDSCs increased the immunosuppressive function of endogenous mMDSCs while decreased the proportions of IL-6^+^ T cells and IFN-γ^+^ CD4^+^ T cells. In addition, the deposition of amyloid plaque decreased while the number of neurons increased in the hippocampus and cortex region after the supplement of exogenous mMDSCs. Furthermore, the number of microglia increased with an increase in the proportion of M2 phenotype.

**Conclusions:**

Pg can reduce the proportion of mMDSCs, induce immune-overreaction, and exacerbate the neuroinflammation and cognitive impairment in 5xFAD mice. Supplement of exogenous mMDSCs can reduce the neuroinflammation, immune imbalance and cognitive impairment in 5xFAD mice infected with Pg. These findings indicate the mechanism of AD pathogenesis and Pg-mediated promotion of AD, and provide a potential therapeutic strategy for AD patients.

**Supplementary Information:**

The online version contains supplementary material available at 10.1186/s12974-023-02743-8.

## Introduction

Alzheimer’s disease (AD) is the most common type of dementia among the elderly worldwide [1]. It is a neurodegenerative disease marked by amyloid plaque deposition and intracellular neurofibrillary tangles in the brain, resulting in the progressive damage of cognitive function [2]. Amyloid plaques are formed by abnormal accumulation of beta-amyloid (Aβ) peptides which can induce neuronal damage or even loss. In addition, activation of neuroinflammation, such as activation of microglia, is a common neuropathological feature of AD. So far, no effective treatment has appeared other than long-term oral medication for symptomatic treatment [3].

There is strong association between AD and periodontitis which is a chronic infectious and inflammatory disease characterized by irreversible attachment loss of involved teeth [4]. *Porphyromonas gingivalis* (Pg) is a keystone pathogen in periodontitis [5] and is also a significant risk factor for various systemic diseases including rheumatoid arthritis [6, 7], ischemic stroke [8] and AD [9]. In the case of AD, periodontitis is associated with more severe cognitive impairment [10]. Besides, Pg and its virulence factors have been found in the postmortem brains of AD patients [11, 12]. Pg can increase the deposition of Aβ in the brain and drive microglia to migrate to the infection site, initiating excessive inflammatory and immune responses in AD [13]. Our recent study demonstrates that Pg-induced cognitive impairment is associated with gut dysbiosis, neuroinflammation, and glymphatic dysfunction [14]. Gingipain is known as a vital virulence factor of Pg, and the small-molecule inhibitors of gingipain can reduce Pg amount, Aβ deposition, neuroinflammation and neuronal loss in the hippocampus region of wild-type mice infected with Pg [12]. It is unclear how Pg and gingipain involve in neuroinflammation and cognitive impairment in AD.

Our previous study indicates that Pg induces periodontitis, causes immune imbalance, and promotes rheumatoid arthritis [7]. It is unknown whether Pg and gingipain promote AD similarly through impairing key immune cells important for immune homeostasis. It is reported that the proportion and suppressive function of myeloid-derived suppressor cells (MDSCs), a heterogeneous population of immature myeloid cells with immune suppressive function, are ascended in prodromal AD patients while descended in those with advancing AD [15]. When compared to patients with mild cognitive impairment, mild AD patients demonstrate severer symptoms and decreased proportion of granulocytic MDSCs (gMDSCs). However, this study is lack of cell functional assay [16]. Whereas Pg can increase both monocytic MDSCs (mMDSCs) and gMDSCs in wild-type mice, this bacterial infection particularly affects mMDSCs [17]. It remains unclear whether Pg can change the proportion and suppressive function of mMDSCs in AD. In the current study, we observed that more severe cognitive impairment and decreased proportion of mMDSCs in 5xFAD mice orally infected with Pg. Further experiments in vitro showed that Pg decreased the proportion and suppressive function of mMDSCs. Finally, exogenous mMDSCs were sorted from wild-type mice and injected into 5xFAD mice infected with Pg, to investigate whether supplement of exogenous mMDSCs could reduce cognitive impairment, neuropathology and immune imbalance induced by oral Pg infection.

## Materials and methods

### Animals and PCR genotyping

Familial Alzheimer's disease (FAD) is caused by mutations in the genes for amyloid precursor protein (APP) and presenilins (PS1, PS2). 5xFAD mice are APP/PS1 double transgenic mice that contain five FAD mutations, and have been reported as an important animal model of AD researches due to the production of high level of Aβ42 and rapid formation of amyloid plaque in brain. In 5xFAD brain, intraneuronal Aβ42 accumulates at 1.5 months and amyloid deposition begins at 2 months with gliosis. At 4–5 months of age, mice show significant neuronal loss and brain dysfunction. Hemizygote can survive and be used for experiments [18].

3-month-old 5xFAD mice were purchased from Guangdong Laboratory Animals Monitoring Institute (Guangzhou, China). All animals were randomly divided into two groups: the control and Pg group (*n* = 6). The experimental procedures were approved and performed according to the guidelines of the Institutional Animal Care and Use Committee of Sun Yat-sen University (Approved No. 2021-000676).

Two parental male 5xFAD mice were generously provided by Professor Weijie Lin of Sun Yat-sen University, Guangzhou, China. Each male 5xFAD mouse was mated with two or three female wild-type mice and produced offspring. All mice used were C57BL/6 background and were maintained under specific pathogen-free conditions in the facility at the Sun Yat-sen University Animal Center (Guangzhou, China), ad libitum food and water (Approved No. 2021-000673).

Tail snips (0.5 cm) of the offspring were collected for genotyping on day 17 post date of birth. Each sample was added in 180 µL tissue digestion buffer (NaOH 50 mM and EDTA 0.2 mM) and heated for 15 min at 100 °C. Following digestion, 20 µL neutralization buffer (1 M Tris buffer, pH 8.0) was added. Samples were centrifuged at 12,000 rpm for 10 min, and 1 µL of the supernatant was used for PCR [19]. For PCR, 10 µL of 2 × Taq PCR MasterMix (Takara, Japan) and 0.4 µL of APP, PS1, and Reference primers (10 μM; Tsingke Biotech, China) were added in each PCR tube. Finally, 1 µL of genomic DNA supernatant and 6.6 µL of nuclease-free water (Solarbio, China) were added to each tube for a final reaction volume of 20 μL. PCR was performed using a standard thermocycler (ABI, USA). The PCR conditions were set as follows: initial denaturation (94 °C for 3 min); followed by 35 cycles of denaturation (94 °C for 30 s), annealing (56 °C for 60 s), and extension (72 °C for 60 s); and a final extension at 72 °C for 5 min. PCR products were removed from the thermocycler and maintained at 4 °C before loading on 2% agarose gels (Biofroxx, Germany). PCR primer sequences involved were listed in Table 1.

**Table 1:** Primers for mouse genotyping

Gene	Forward primer (5’ → 3’)	Reverse primer (5’ → 3’)
APP	AGGACTGACCACTCGACCAG	CGGGGGTCTAGTTCTGCAT
PS1	AATAGAGAACGGCAGGAGCA	GCCATGAGGGCACTAATCAT
Reference	CTAGGCCACAGAATTGAAAGATCT	GTAGGTGGAAATTCTAGCATCATCC

Partial results of mouse genotyping were demonstrated (Additional file 1: Figure S1). In this study, 4-month-old female 5xFAD mice were used. All animals were randomly assigned to three groups: the control, Pg and Pg plus mMDSCs group (*n* = 5). The experimental procedures were approved and performed according to the guidelines of the Institutional Animal Care and Use Committee of Sun Yat-sen University (Approved No. 2021–000,676).

### Oral infection of Pg

The culture of Pg was performed as previously described [20]. Briefly, Pg (strain W83) was cultured in brain heart infusion medium (HuanKai Microbial, China) supplemented with 1% yeast extract (OXOID, UK), 0.001% menadione (Sigma, USA) and 0.5% hemin (Solarbio, China) at 37 °C for two days in an anaerobic atmosphere in use of an AnaeroPack (C-1; MGC, Tokyo, Japan). Then, the bacteria were harvested, centrifuged, and washed with phosphate buffered saline (PBS). The number of Pg was estimated by measurement of the optical density at 600 nm (OD_600_).

Live Pg was suspended in a total of 1.0 × 10^9^ colony-forming units (CFU) in 0.1 mL PBS with 2% carboxymethyl cellulose (CMC) (Solarbio). This suspension was supplied to each mouse by oral gavage three times a week for 1 month, or oral administration every other day for 6 weeks. The control group was administered 0.1 mL PBS with 2% CMC without Pg.

### Cell preparation

The single-cell preparation was obtained as previously described [21]. Briefly, peripheral blood, spleen, bone marrow and mesenteric lymph nodes (MLN) of 5xFAD mice treated with or without Pg were harvested. Tissues of spleen and MLN were gently ground and filtered through 40 μm sterile cell filters, respectively. Femurs and tibias were collected, bone marrow tissues were flushed out of the bone marrow cavity with PBS. Erythrocytes of blood, spleen and bone marrow were lysed using RBC lysis buffer (CWBIO, China) according to the manufacturer’s instructions, and then, the remaining cells were washed with PBS for twice.

### Flow cytometry

Single-cell preparation of each tissue was incubated with anti-mouse CD16/32 Fc block (BioLegend, USA) and LIVE/DEAD™ Fixable Near-IR Dead Cell Stain Kit (Invitrogen, USA) on ice in the dark for 15 min. Then, the cells were washed with PBS and stained with surface staining antibodies on ice in the dark for 20 min. For intracellular staining, the cells were stimulated with 10 μg/mL lipopolysaccharide (LPS), 50 ng/mL phorbol 12-myristate 13-acetate (PMA), 500 ng/mL ionomycin (Sigma) and 2 μM monensin (eBioscience, USA) for 5 h before surface staining. Cytofix/Cytoperm Plus Fixation/Permeabilization Solution Kit (BD, USA) was utilized prior to incubation with intracellular antibodies. The following antibodies purchased from Biolegend or Invitrogen were used for flow cytometry: CD11b APC or eF506, Gr1 PC5 or FITC, Ly6C PE, Ly6G PC7 or SB600, CD4 PE/Dazzle, CD8 AF700, CD19 SB780, IL-10 APC, and CD25 PC7. All data were collected on Fortessa (BD) or Cytoflex (Beckman, USA) and analyzed with FlowJo software (TreeStar, version X, USA).

### MDSCs culturing and stimulation

Single-cell preparation of blood, spleen and bone marrow were plated at 2 × 10^6^ cells/mL and cultured in complete RPMI-1640 medium supplemented with 2 ng/mL GM-CSF (PrimeGene, China). Pg was added at indicated multiplicities of infection (MOI = 10) for 48 h. Afterward, the cells could be analyzed by flow cytometry or be stimulated for further use.

The cells of bone marrow were stimulated with 0.1 μg/mL LPS (Sigma) and 100 U/mL IFN-γ (Novoprotein, China) overnight before T cell proliferation assay and mMDSCs injection [22].

### T cell proliferation assay

Single-cell preparation from MLN of wild-type mice was labeled with Cell Proliferation Dye eFluor™ 450 (eBioscience) according to the manufacturer’s protocol. Then, the labeled cells of MLN were co-cultured with the stimulated cells of bone marrow from 5xFAD mice at a ratio of 1:1 in the presence of plate-bound anti-mouse CD3 (5 μg/mL; Biolegend) and soluble anti-mouse CD28 antibodies (1 μg/mL; Biolegend) in complete RPMI-1640 medium for 72 h. The intensity of Dye eFluor™ 450 fluorescence was analyzed to determine the proliferation of T cells by flow cytometry.

CD4^+^ T cells were sorted from MLN of wild-type mice using anti-mouse CD4^+^ antibody and PE microbeads (Stemcell, USA). CD4^+^ T cells were then labeled with Cell Proliferation Dye eFluor™ 450, and co-cultured with sorted mMDSCs or gMDSCs at a ratio of 1:1 in 96-well plates in the presence of anti-CD3 and anti-CD28 antibodies for 72 h [23]. The intensity of Dye eFluor™ 450 fluorescence was analyzed to determine the proliferation of CD4^+^ T cells by flow cytometry.

### Cell sorting, stimulation and injection

Single-cell suspension prepared from bone marrow was blocked with anti-mouse CD16/32 Fc block. Then, the cells were stained with anti-mouse CD11b APC, Gr1 FITC, Ly6C PE antibodies (Biolegend) on ice in the dark for 20 min. After that, cells were washed with PBS and suspended in PBS supplemented with 2% fetal bovine serum (Hyclone, USA). CD11b^+^, Gr1^+^ and Ly6C^+^ populations were sorted as mMDSCs while CD11b^+^, Gr1^+^ and Ly6C^−^ populations were sorted as gMDSCs using a cell sorter (BD FACSAriaFusion, USA).

The sorted cells were cultured in complete RPMI-1640 medium with 0.1 μg/mL LPS and 100 U/mL IFN-γ overnight for stimulation.mMDSCs were washed twice and suspended in PBS on ice. Each mouse of Pg plus mMDSCs group was injected with 100 µL of 1 × 10^6^ cells through tail vein using 1 mL syringes per week for 4 weeks. Each mouse of control group and Pg group was injected with 100 µL PBS.

### Morris water maze test

Morris water maze (MWM) test was performed in accordance to the protocol previously described [24]. The maze was made up of a circular pool (120 cm in diameter, 50 cm in height) with a white escape platform (10 cm in diameter) located 1 cm below the surface of opaque water. MWM contained 5-day place navigation training and subsequent spatial probe test. Mice were placed into the maze from four quadrants of the pool every day for five consecutive days. The escape latency would be recorded when mice located and climbed onto the platform. The mice were guided to the platform and placed on it for 10 s manually if failing to find the platform within 60 s. On the sixth day, the platform was removed. Mice were placed into the quadrant opposite to the original location of platform and freely swam for 60 s. The swimming traces were recorded by an overhead camera and tracked by automated software (San Diego Instruments, San Diego, CA, USA).

### Open field test

Open field test (OFT) was carried out for each mouse before sacrifice. The open field consisted of a blue square box (45 × 45 × 45 cm). For habituation, all mice were first placed in the central area of the box for 5 min. Then, the mice were allowed to freely explore for 10 min in OFT [25]. Locomotor activity of mice was video-recorded by a fixed camera overhead and processed by software (TopScan—TopView Behavior Analyzing system). The box was cleaned after each trial.

### Immunofluorescent staining

The coronal brain slices were blocked with 5% (w/v) BSA and 0.1% Triton X-100 (Sigma) in PBS at 37 °C for 1 h. The slices were subsequently incubated with primary antibodies at 4 °C overnight, including mouse anti-Aβ_1–42_ (BioLegend), mouse anti-NeuN (Abcam, UK), rabbit anti-Iba1 (Wako, Japan), mouse anti-CD206 (Abcam). The following day, the sections were incubated with secondary antibodies (Cell Signaling Technology, USA) for 1 h at room temperature. The number of cells were measured by two individuals in a double-blind manner with ImageJ software (version 1.46r, USA).

### Quantitative reverse transcription polymerase chain reaction (qRT-PCR)

Total RNA was extracted from the splenocytes and the cells of bone marrow with TRNzol reagent (TIANGEN, China) and acquired RNA was quantified on Nanodrop 2000. 1 μg of total RNA was treated by the cDNA Synthesis Kit (Yeasen, Biotech, China) for reverse transcription based on the manufacturer’s instructions. qRT-PCR was carried out using the SYBR Green Kit (Yeasen, Biotech) on QuantStudio 5 (ABI). The primers involved were listed in Table 2.

**Table 2:** Primers for qRT-PCR

Primers	Forward primer (5’ → 3’)	Reverse primer (5’ → 3’)
GAPDH	AGGTCGGTGTGAACGGATTTG	GGGGTCGTTGATGGCAACA
Arg1	CATTGGCTTGCGAGACGTAGAC	GCTGAAGGTCTCTTCCATCACC
Nos2	GAGACAGGGAAGTCTGAAGCAC	CCAGCAGTAGTTGCTCCTCTTC

### Data analysis

All data were analyzed with GraphPad Prism 9. The difference in measurement data between two groups was assessed using a *t* test for normal descriptive data and nonparametric Mann–Whitney test for nonnormal descriptive data. For analysis of the MWM test and body weight, two-way repeated measures ANOVA with Sidak’s test for multiple comparisons were performed. Data were expressed as means ± SEM and *p* value < 0.05 was considered statistically significant.

## Results

### Oral Pg infection reduced the proportion of mMDSCs and exacerbated cognitive impairment and neuroinflammation in 5xFAD mice

**Fig. 1:**
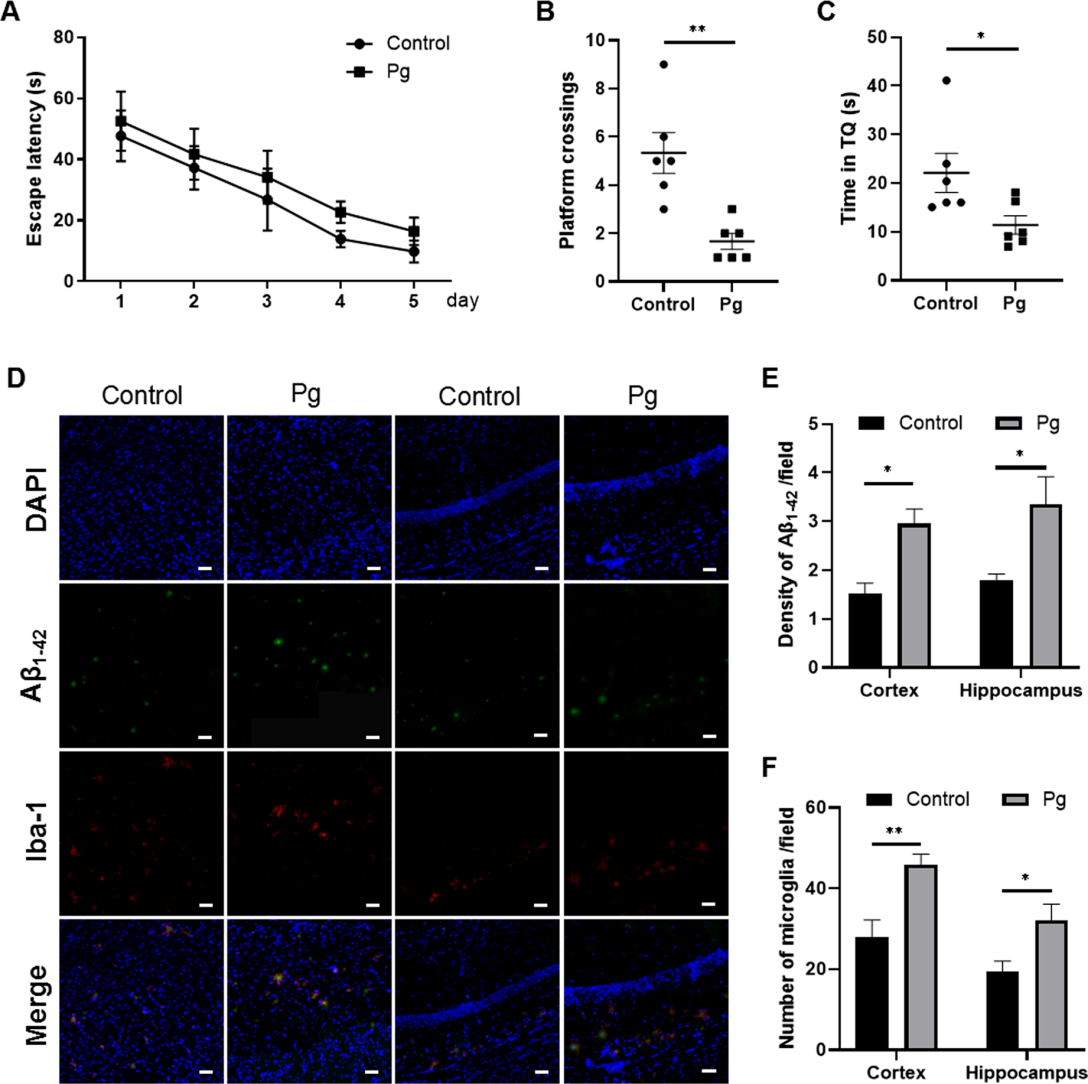
Pg exacerbated cognitive impairment and neuroinflammation in 5xFAD transgenic mice. **A** Escape latencies of the two groups of mice in the spatial acquisition trial of the Morris water maze (MWM). **B** Times of mice crossing over the platform in the probe trial of the MWM. **C** Time of mice spent in the target quadrant (TQ) in the probe trial of the MWM. **D** Representative images presenting amyloid plaque and Iba-1-positive cells in the cortex and hippocampus regions. **E**, **F** Statistical results of **D** were shown. Data are presented as means ± SEM. * *p* < 0.05; ** *p* < 0.01. Scale bar, 50 μm

We carried out MWM test to evaluate the learning and spatial memory abilities of 5xFAD mice after Pg administration. The results of 5-day place navigation training showed that the escape latency of the Pg group was longer than the control group. Even though there was no significant difference, the longer escape latency of the Pg group during the training period implied a worse spatial learning ability to some extent (Fig. 1A). The result of probe trial verified the impairment of spatial memory ability in the Pg group. The number of times of mice crossing the area where the original platform located was significantly decreased in Pg-treated mice compared to the control group (Fig. 1B). Besides, mice infected with Pg spent much less time in the target quadrant (TQ) compared with the control ones (Fig. 1C). Taken together, our results indicated that Pg infection induced a decline in spatial cognition of 5xFAD mice.

We analyzed the deposition of amyloid plaque (Aβ_1–42_) and the activation of microglia (Iba-1) in the cortex and hippocampus regions after Pg administration by immunofluorescent staining (Fig. 1D). We found that amyloid plaque was significantly increased in the Pg group in the cortex and hippocampus regions (Fig. 1E). In addition, Pg significantly increased the number of microglia in the hippocampus and cortex regions compared to the control group (Fig. 1F). These results demonstrated that Pg infection exacerbated neuroinflammation in 5xFAD mice.

**Fig. 2:**
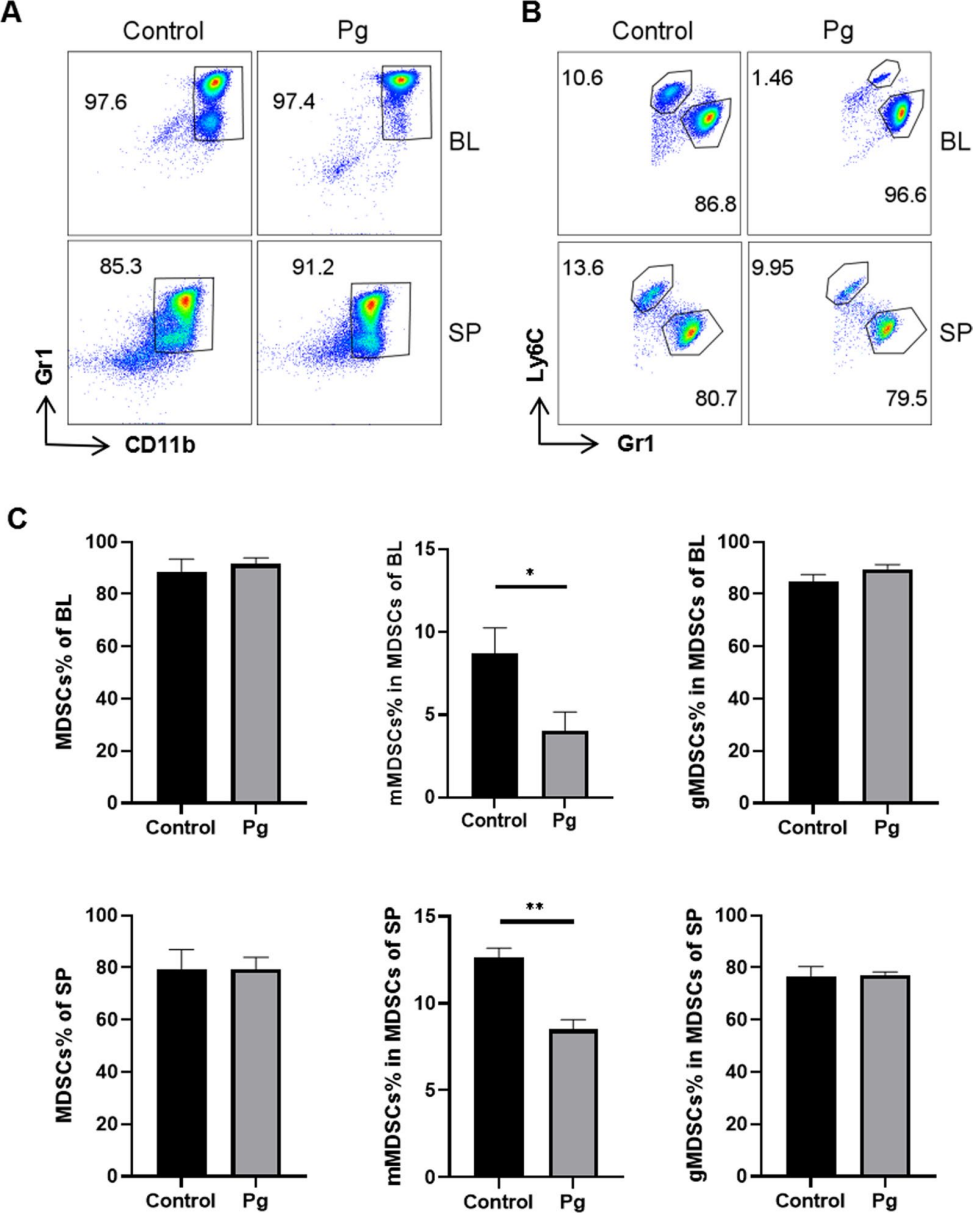
Pg reduced the proportion of mMDSCs in 5xFAD mice. Representative FACS plots of MDSCs (**A**), mMDSCs marked as Gr1^lo^Ly6C^hi^ and gMDSCs marked as Gr1^hi^Ly6C^lo^, (**B**) in blood (BL) and spleen (SP) of 5xFAD mice with or without Pg gavage for 4 weeks. **C** Statistical results of (**A**, **B**) were shown respectively. Data are presented as means ± SEM. * *p* < 0.05; ** *p* < 0.01

MDSCs, gMDSCs and mMDSCs from peripheral blood and spleen of 5xFAD mice were stained with specific antibodies and analyzed by flow cytometry, the gating strategies and representative FACS plots are shown in Fig. 2A, B. The proportions of MDSCs and gMDSCs did not change in blood and spleen of 5xFAD mice with or without Pg administration, while the proportion of mMDSCs was significantly decreased in blood and spleen after Pg infection (Fig. 2C).

### Pg decreased the proportion and immunosuppressive function of mMDSCs in 5xFAD mice in vitro

**Fig. 3:**
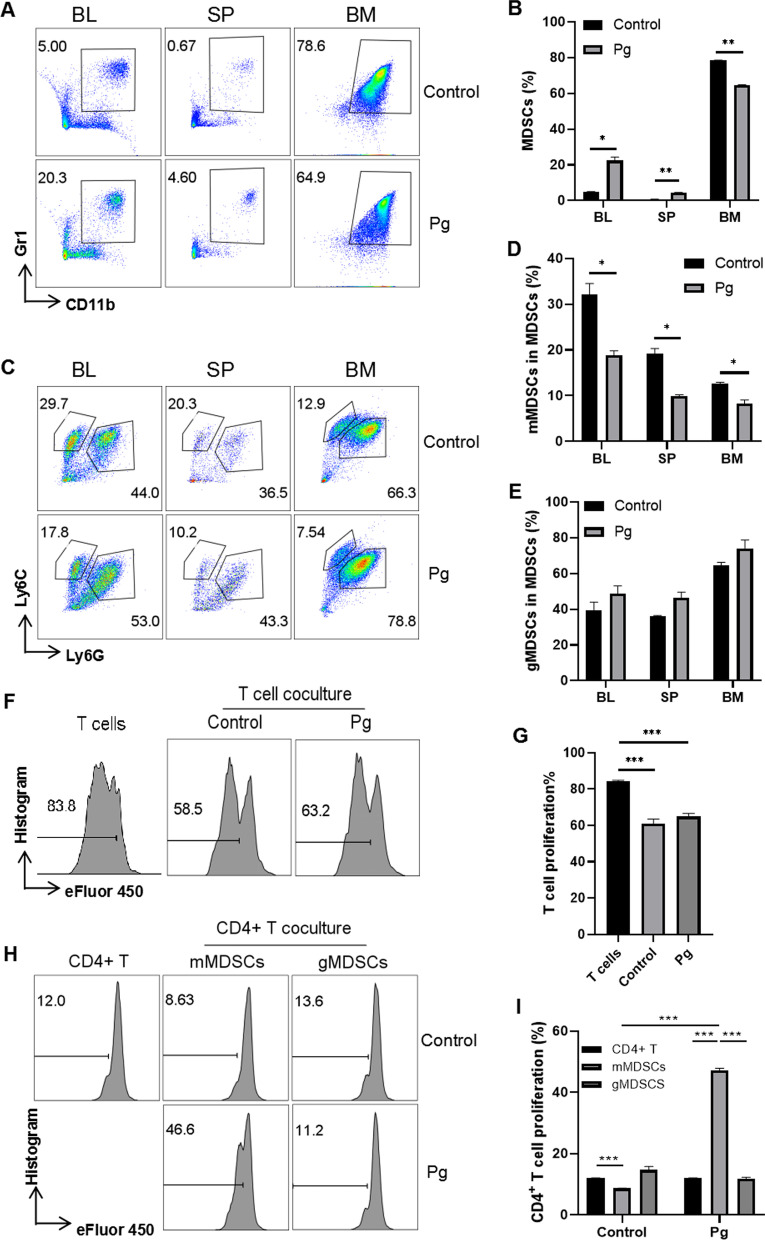
Pg reduced the proportion and immunosuppressive function of mMDSCs in vitro. Representative FACS plots of gating strategy for MDSCs (**A**) and two subpopulations (**C**) in BL, SP, and bone marrow (BM) of 5xFAD mice with or without Pg treatment. The proportions of MDSCs (**B**), mMDSCs (**D**) and gMDSCs (**E**) were presented. **F** Representative FACS plots of proliferation of MLN cells (mainly T cells) labeled with eFluor 450 and co-cultured with BM cells for 48 h. **G** Statistical results of **F**. **H** Representative FACS plots of eFluor 450 labelled CD4^+^ T cells sorted from MLN cells and co-cultured with sorted mMDSCs and gMDSCs for 48 h. **I** Statistical results of **H**. Data are presented as means ± SEM. * *p* < 0.05; ** *p* < 0.01; *** *p* < 0.001

Leukocytes from peripheral blood, spleen and bone marrow of 5xFAD mice were co-cultured with Pg for 48 h in vitro, and then, we detected the proportional changes of MDSCs, gMDSCs and mMDSCs by flow cytometry. The gating strategies of MDSCs, gMDSCs and mMDSCs are shown in Fig. 3. The proportions of MDSCs were significantly increased in peripheral blood and spleen while decreased in bone marrow after Pg infection (Fig. 3B). The proportions of mMDSCs were significantly decreased in blood, spleen and bone marrow after Pg infection (Fig. 3D). For gMDSCs, there was no significant difference, even though an uptrend was observed (Fig. 3E).

Regarding the immunosuppressive function, single-cell preparation of MLN was co-cultured with Pg-treated bone marrow cells to check the overall functional changes, since bone-marrow cells mainly consist of MDSCs (60–80%) and the majority of MLN cells are CD4^+^ and CD8^+^ T cells (> 90%). The T cell proliferation was significantly inhibited by bone-marrow cells while slightly increased after Pg treatment (Fig. 3F, G). To further examine the immunosuppressive function of mMDSCs and gMDSCs in T cell proliferation, mMDSCs and gMDSCs were sorted by flow cytometry and co-cultured with the sorted CD4^+^ T cells. The results showed that the proliferation of CD4^+^ T cell was suppressed by mMDSCs but not gMDSCs (Fig. 3H, I). Interestingly, the proliferation of CD4^+^ T cell increased when co-cultured with Pg-treated mMDSCs but not gMDSCs (Fig. 3H, I). Taken together, Pg decreased the proportion and T-cell-suppressive function of mMDSCs of 5xFAD mice in vitro.

### Exogenous supplement of mMDSCs improved behavioral function damaged by Pg in 5xFAD mice

**Fig. 4:**
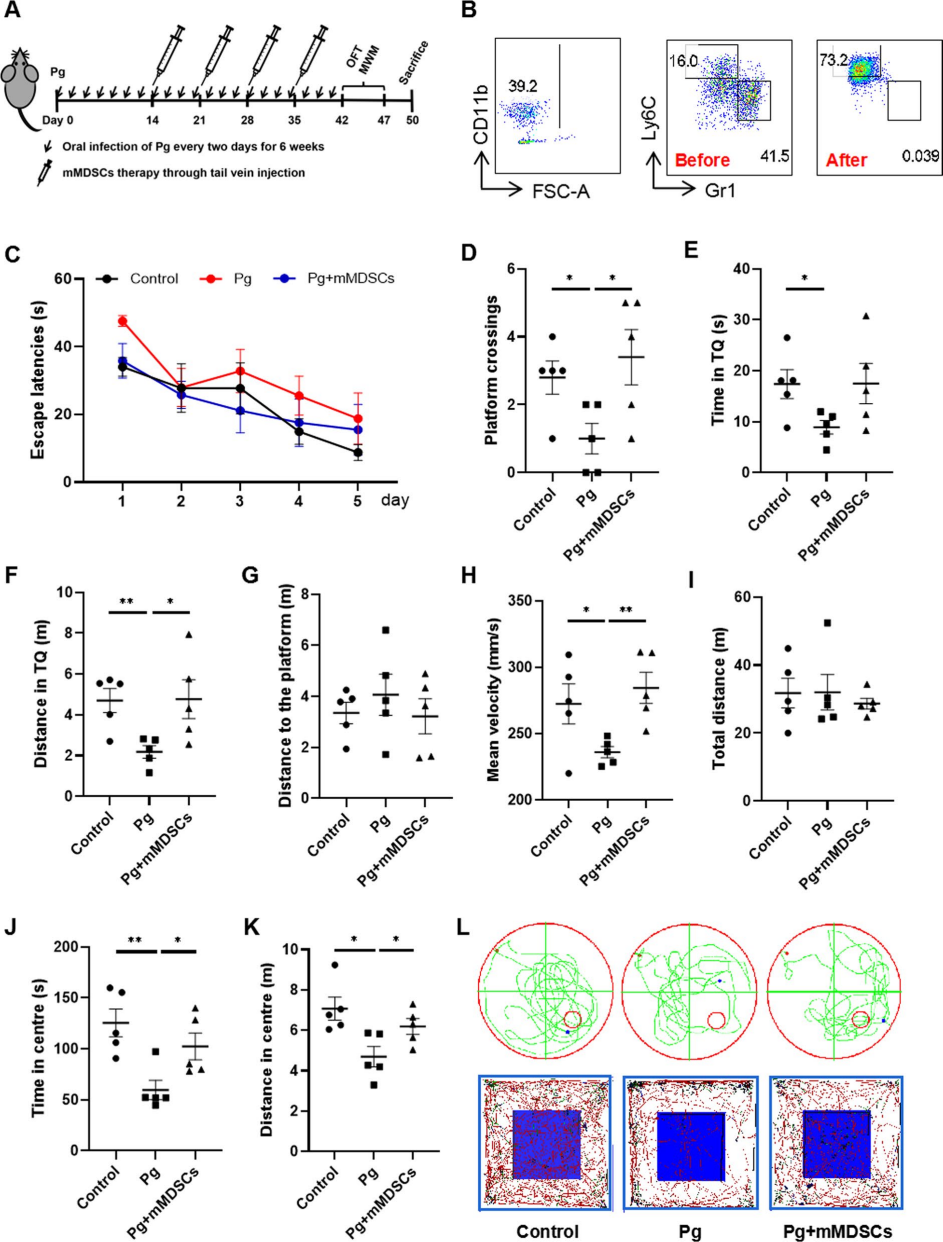
Exogenous mMDSCs improved behavioral function of 5xFAD mice infected with Pg. **A** Experiment design of oral Pg administration and intravenous mMDSCs injection in 5XFAD mice. **B** Representative FACS plots of gating strategy for mMDSCs sorting with BM cells and the sorting efficiency. **C** Escape latencies of three groups of mice in the spatial acquisition trial of the MWM. **D**Times of mice crossing over the platform area in the probe trial of the MWM. **E**, **F** Time and travelling distance of mice in the TQ in the probe trial of the MWM. **G** Distance travelled to the platform area in the probe trial of the MWM. **H** Average swimming speed of mice in the probe trial of the MWM. **I** Total distance travelled in the open field test (OFT). **J** Moving distance in the central area in the OFT. **K** Time spent in the central area in the OFT. **L** Representative traces of each group in the MWM and OFT. Data are presented as means ± SEM. * *p* < 0.05; ** *p* < 0.01

5xFAD mice were orally administrated with Pg for 6 weeks and mMDSCs injection began after 2 weeks of Pg infection (Fig. 4A). The gating strategy of mMDSCs sorting and representative FACS plots before and after the cell sorting were presented (Fig. 4B). There was no significant difference in the body weight among the three groups of 5xFAD mice (Additional file 1: Figure S2). In the MWM test, the escape latency of the Pg group was longer than the control and Pg plus mMDSCs group during the 5-day training, though no significant difference was observed. In addition, mMDSCs injection seemed to partially improve the learning ability impaired by Pg infection (Fig. 4C). In the probe trial, the times of mice crossing the original platform area was significantly reduced in Pg-treated mice compared to the control group and Pg plus mMDSCs group, respectively (Fig. 4D). Mice infected with Pg spent much less time and traveled a shorter distance in the target quadrant compared with the control group. In addition, Pg plus mMDSCs group spent much more time and traveled a longer distance in the target quadrant compared with the Pg group (Fig. 4E, F). Pg-administered mice failed to locate the original platform area and travelled in other quadrants, while mice with mMDSCs treatment could locate the original platform area more quickly and travelled a longer distance in the TQ (Fig. 4L). There were no significant differences in the distance travelled to the platform among the three groups (Fig. 4G). There was an obvious decrease in swimming speed of the Pg group than that of the control group. In addition, mMDSCs treatment significantly increased the swimming speed compared to the Pg group, suggesting that the motor function and endurance of mice were impaired by Pg infection, while mMDSCs could rescue the corresponding function (Fig. 4H). Taken together, our results indicated that Pg infection induced a decline in spatial cognition of 5xFAD mice and exogenous supplement of mMDSCs could rescue it.

OFT was performed to confirm the changes of spontaneous activity of 5xFAD mice. Compared to the control group, Pg-treated mice mainly stayed at the peripheral area of the box and showed little willingness to explore the central area. The travelling distance and time spent in the central area were both significantly decreased after Pg administration. However, mMDSCs injection increased the travelling distance and time spent in the central area compared to the Pg group (Fig. 4J, K). Representative traces of three groups were presented (Fig. 4L). There was no significant difference in total travelling distance among the three groups (Fig. 4I).

### Exogenous mMDSCs increased the proportion and immunosuppressive function of mMDSCs in 5xFAD mice infected with Pg

**Fig. 5:**
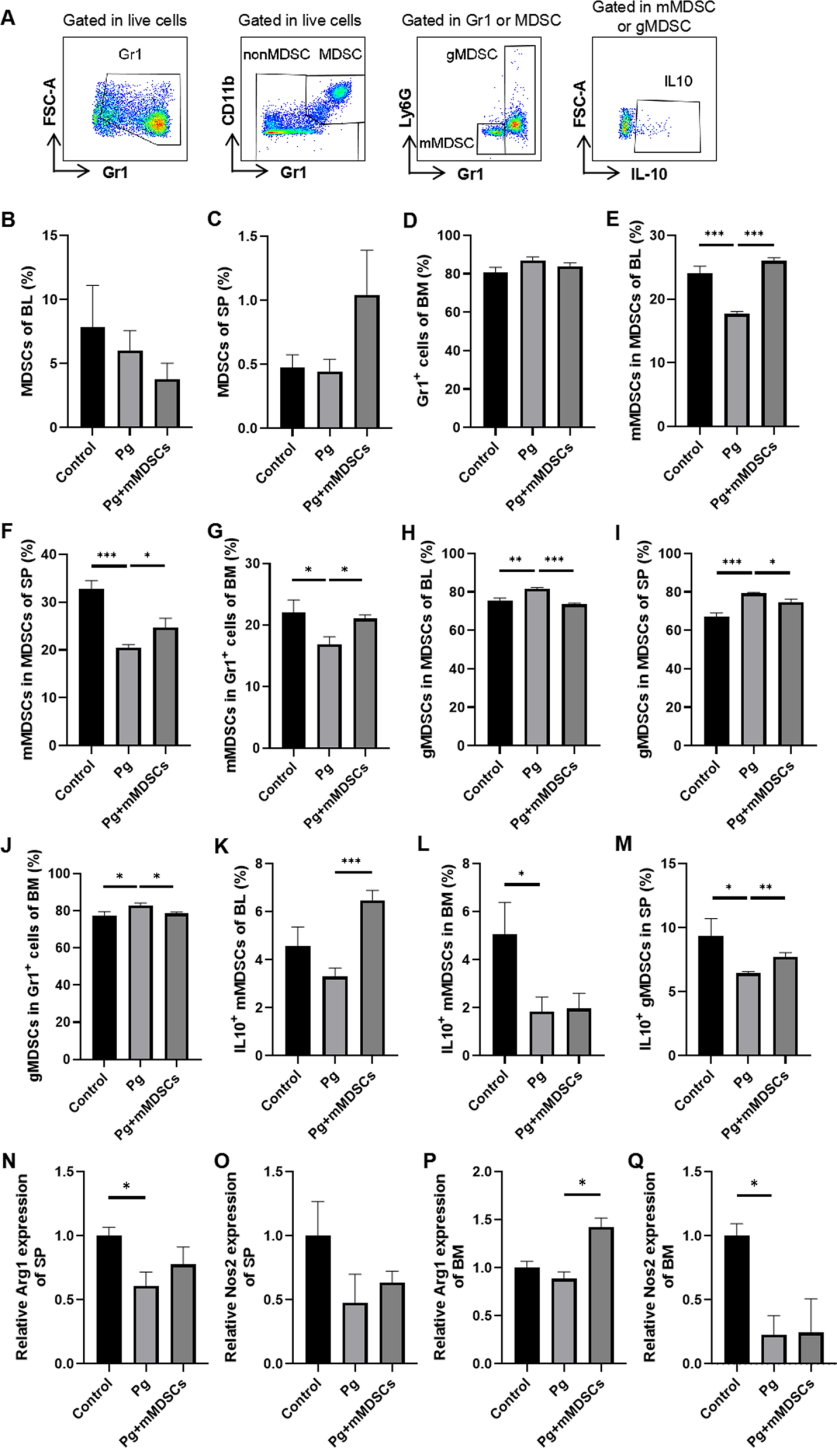
Exogenous mMDSCs increased the proportion and immunosuppression of mMDSCs in 5xFAD mice infected with Pg. **A** Representative FACS plots of gating strategy for Gr1^+^ cells, MDSCs, mMDSCs, gMDSCs and IL-10-producing mMDSCs or gMDSCs. Statistical results of MDSCs proportions in BL (**B**) and SP (**C**). **D** Statistical results of Gr1^+^ cells proportions in BM. Statistical results of mMDSCs proportions in BL (**E**), SP (**F**) and BM (**G**). Statistical results of gMDSCs proportions in BL (**H**), SP (**I**) and BM (**J**). Statistical results of IL-10-producing mMDSCs proportions in BL (**K**) and BM (**L**). **M** Statistical results of IL-10-producing gMDSCs proportions in SP. Relative mRNA expression of Arg1 (**N**) and Nos2 (**O**) in SP detected by qRT-PCR. Relative mRNA expression of Arg1 (**P**) and Nos2 (**Q**) in BM detected by qRT-PCR. Data are presented as means ± SEM. * *p* < 0.05; ** *p* < 0.01; *** *p* < 0.001

As mentioned before, Pg infection decreased the proportion and immunosuppressive function of mMDSCs in 5xFAD mice. To address whether supplement of exogenous mMDSCs could reverse those alterations, we analyzed the proportion and function of MDSCs, mMDSCs and gMDSCs by flow cytometry (Fig. 5A). There were no significant differences in the proportions of MDSCs in peripheral blood and spleen as well as the proportions of Gr1^+^ cells in bone marrow among the three groups (Fig. 5B–D). The proportions of mMDSCs were significantly decreased after Pg infection while significantly increased after exogenous supplement of mMDSCs in peripheral blood, spleen, and bone marrow (Fig. 5E–G). The proportions of gMDSCs were significantly increased after Pg infection while significantly decreased after exogenous supplement of mMDSCs in peripheral blood, spleen, and bone marrow (Fig. 5H–J).

Various mechanisms mediate the immunosuppressive function of mMDSCs, including IL-10 cytokine secretion, the arginase 1 (Arg1) and inducible nitric oxide synthase (iNOS/Nos2) enzymatic activity. Hence, we examined the proportions of IL-10-producing mMDSCs or gMDSCs and the relative expression levels of Arg1 and Nos2 in the spleen and bone-marrow tissue. The proportion of IL-10-producing mMDSCs tended to decrease after Pg infection while significantly increased after exogenous supplement of mMDSCs (Fig. 5K). The proportion of IL-10-producing mMDSCs in bone marrow significantly decreased after Pg infection, while there was no obvious change after exogenous mMDSCs injection (Fig. 5L). The proportion of IL-10-producing gMDSCs in spleen significantly decreased after Pg infection while significantly increased after exogenous supplement of mMDSCs (Fig. 5M). The relative expression level of Arg1 was significantly down-regulated in the spleen of the Pg group and there was a similar trend in the bone marrow. The relative expression of Arg1 was significantly up-regulated in the bone marrow after mMDSCs injection and there was a similar trend in spleen (Fig. 5N, P). In addition, the relative expression level of Nos2 was significantly reduced in bone marrow of the Pg group and there was a similar trend in spleen. However, there were little alterations of Nos2 expression after mMDSCs injection (Fig. 5O, Q). The overall effects of Pg infection on mMDSCs in vivo were consistent with those in vitro, and exogenous supplement of mMDSCs could partially reverse the changes.

**Fig. 6:**
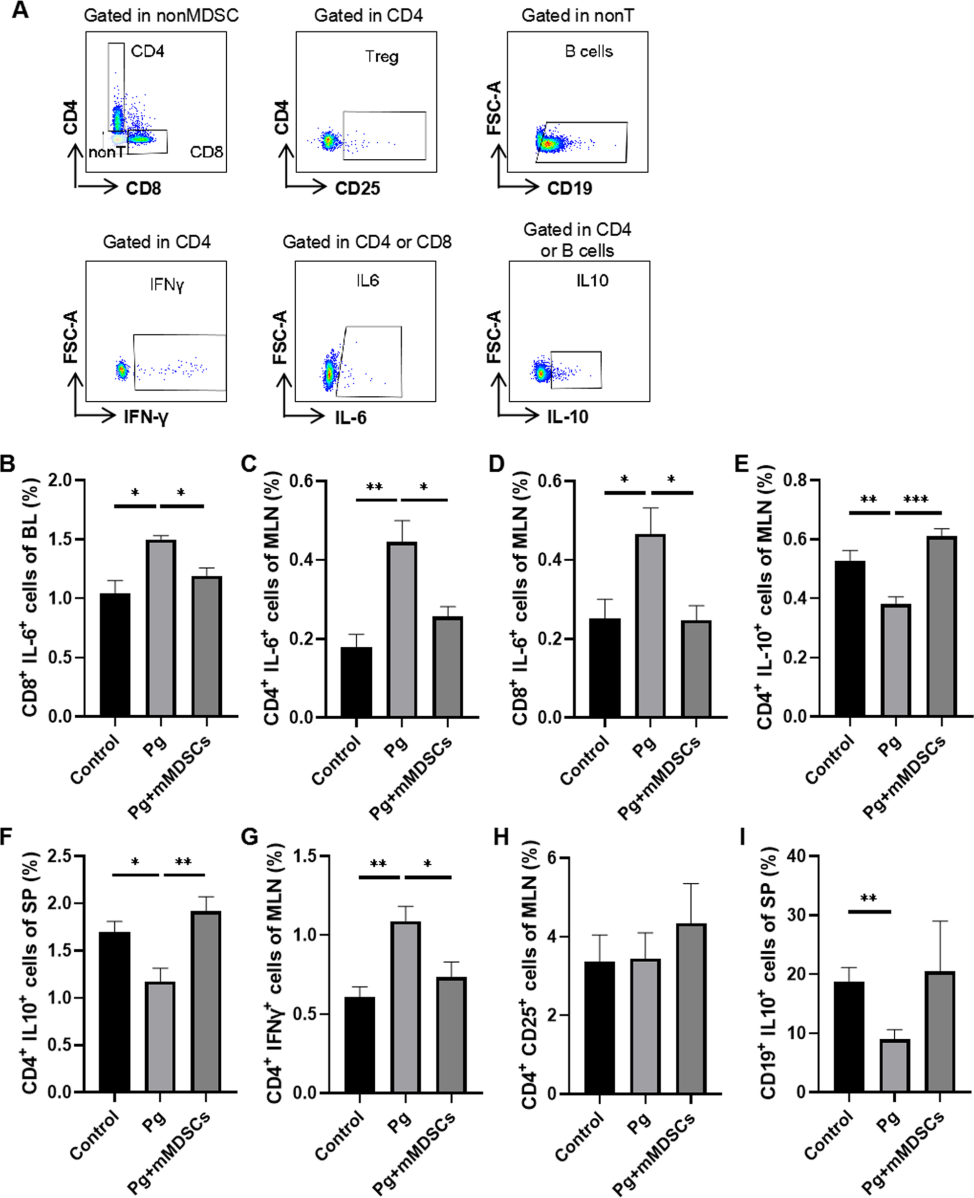
Exogenous mMDSCs ameliorated the immune imbalance in 5xFAD mice infected with Pg. **A** Representative FACS plots of gating strategy of immune cells. **B** Statistical results of CD8^+^ IL-6^+^ cells in BL. Statistical results of CD4^+^ IL-6^+^ cells **(C**) and CD8^+^ IL-6^+^ cells (**D**) in MLN. Statistical results of CD4^+^ IL-10^+^ cells in MLN (**E**) and SP (**F**). Statistical results of CD4^+^ IFN-γ^+^ cells (**G**) and Treg (**H**) in MLN. **I** Statistical results of B10 cells in SP. Data are presented as means ± SEM. * *p* < 0.05; ** *p* < 0.01; *** *p* < 0.001

To explore the influence of exogenous mMDSCs on other immune cells, we tested the changes of immune cells in peripheral blood, spleen and MLN (Fig. 6A). The proportions of CD4^+^ IL-6^+^ and CD8^+^ IL-6^+^ T cells in MLN tissue increased after Pg infection while decreased after mMDSCs injection (Fig. 6B, C). The proportion of CD8^+^ IL-6^+^ T cells in peripheral blood also increased after Pg infection while decreased after mMDSCs injection (Fig. 6D). In addition, the proportion of CD4^+^ IFN-γ^+^ T cells in MLN tissue increased after Pg infection while decreased after mMDSCs injection (Fig. 6G). The proportions of CD4^+^ IL-10^+^ T cells in MLN and spleen tissue decreased after Pg infection while increased after mMDSCs injection (Fig. 6E, F). There were no significant differences in the proportions of Treg among the three groups (Fig. 6H). The proportion of B10 cells in spleen decreased significantly after Pg infection while increased after mMDSCs injection (Fig. 6I).

### Exogenous mMDSCs reduced the neuroinflammation in 5xFAD mice infected with Pg

**Fig. 7:**
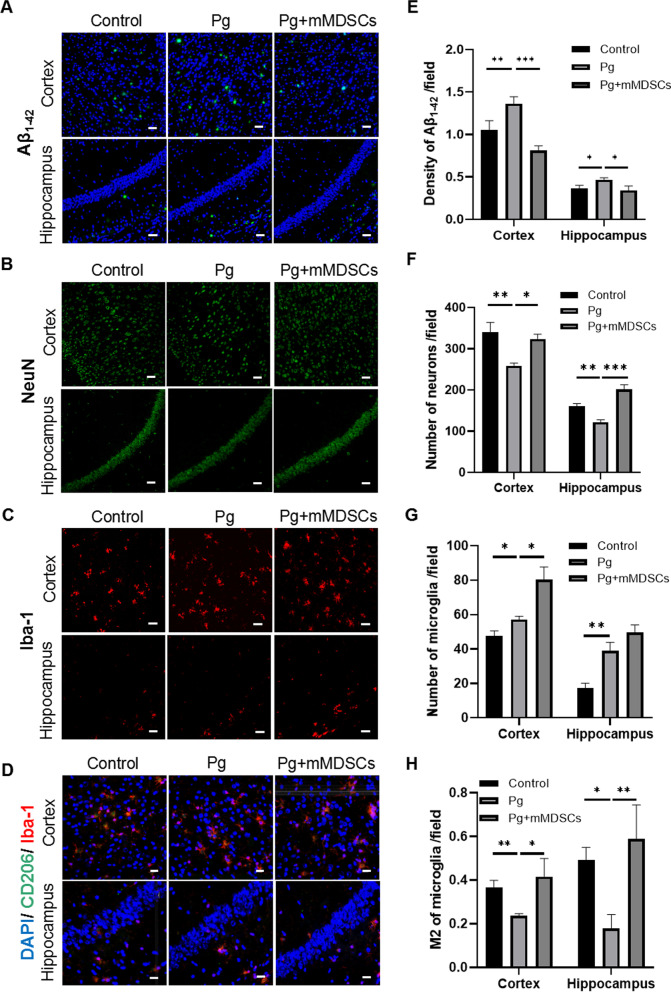
Exogenous mMDSCs reduced the neuroinflammation in 5xFAD mice infected with Pg. Representative images presenting amyloid plaque (**A**), NeuN (**B**), Iba-1-positive cells (**C**), Iba-1 and CD206-positive cells (**D**) in the cortex and hippocampus regions. **E**–**H** Statistical analysis corresponding to (**A**–**D**). Data are presented as means ± SEM. * *p* < 0.05; ** *p* < 0.01; *** *p* < 0.001. Scale bar, 50 μm

Amyloid plaque deposition was significantly increased in the Pg group and decreased in the Pg plus mMDSCs group in the cortex and hippocampus regions (Fig. 7A, E). Moreover, the number of neurons was significantly reduced in Pg-infected mice while increased in mMDSCs-injected mice both in the cortex and hippocampus regions (Fig. 7B, F).

Pg significantly increased the number of microglia in the cortex and hippocampus regions compared to the control group. Interestingly, the number of microglia also increased after exogenous supplement of mMDSCs (Fig. 7C, G). To further explore the properties of microglia, we detected the ratio of M2 phenotype. The proportions of M2 phenotype were significantly decreased in the Pg group while increased after mMDSCs injection in the cortex and hippocampus regions (Fig. 7D, H). These data suggested that Pg increased the Aβ deposition, neuronal loss and decreased the M2-phenotype microglia. In addition, more importantly, exogenous supplement of mMDSCs ameliorated the brain pathology caused by Pg infection.

## Discussion

In this study, we found that oral Pg exacerbated cognitive impairment and neuroinflammation of 5xFAD mice. In addition, the proportion and immunosuppressive function of mMDSCs were decreased after Pg infection in vivo and in vitro. However, exogenous supplement of mMDSCs enhanced the cognitive function impaired by Pg infection, and ameliorated immune imbalance and neuroinflammation in 5xFAD mice.

### Contribution of Pg in the association of periodontitis and AD

Pg is a Gram-negative bacteria, and is tightly associated with the occurrence and development of AD [26]. Cognitive impairment is recognized as an early symptom of AD. There is a strong association between Pg and impairment of cognitive function in a direct or indirect manner. It is estimated that patients with severe periodontitis swallow about 10^12^ ~ 10^13^ Pg per day [27]. Pg-LPS and gingipains may lead to the accumulation of Aβ and have been directly detected in the brains of AD patients [11, 12]. Pg can enter the brains of ApoE^−/−^ mice and thereby promote the neuronal injury [24]. Besides, Pg-LPS can induce cognitive impairment mediated by neuroinflammation in wild-type mice [28]. On the other hand, Pg can prompt Aβ production of peripheral macrophages which may in turn increase the cerebral Aβ deposition [29]. Besides, our group previously discovered that Pg could induce dysbiosis of gut microbiota and then result in cognitive impairment and pathological changes of brain through the gut-brain axis in 9–10-month-old wild-type mice [14]. In this study, the results of the WMW test and OFT showed that oral Pg infection exacerbated AD-like behaviors of 5xFAD mice. This indicated that Pg could impair the spatial cognition and spontaneous activity of 5xFAD mice, which was mediated by the increased Aβ deposition and the decreased number of neurons in the brains.

### Characterization of MDSCs in the Pg-mediated promotion of AD

Oral Pg infection is reported to strongly related to periodontitis, and the resulting tissue destruction contributes to host immune responses [30]. Evidences have illustrated that MDSCs play a vital part in inflammatory immune responses due to bacterial infection [31]. Both subpopulations of MDSCs are CD11b^+^ Gr1^+^, and myeloid differentiation antigen Gr1 contains two epitopes defined as anti-Ly6C and anti-Ly6G antibodies. CD11b^+^ Gr1^low^ cells (mostly mMDSCs) show more immunosuppressive than CD11b^+^ Gr1^hi^ cells (mostly gMDSCs) [32]. Compared to gMDSCs, mMDSCs appear a higher suppressive ability by producing IL-10 and NO [33]. Infection with Pg induces the expansion of both total and subpopulations of MDSCs in the bone marrow and spleen among 8-week-old wild-type mice, of which only mMDSCs could strongly suppress the proliferation of CD4^+^ T cells [17]. The 3-month-old 5xFAD mice used in this study might have different situation. A previous study demonstrates that MDSCs are increased in blood in healthy elderly individuals compared with young individuals [34]. Therefore, the frequency and function of MDSCs might be closely associated with age. Further studies are urgently needed to clarify this issue. Besides, a clinical research reports that the number and immunosuppressive function of MDSCs in blood are ascended in prodromal AD but are rapidly descended in advancing AD. In fact, despite an increase in number, the suppressive function of MDSCs has already begun to decline in mild AD [15]. Another clinical study documents an increase in total MDSCs in blood, especially in gMDSCs of amnestic mild cognitive impairment patients but not of mild AD patients and healthy people. However, the percentages of mMDSCs are not different among three groups [16]. Since inflammatory responses are still powerful in prodromal AD, the increasing number of MDSCs may seem to be a way of compensation. Nevertheless, increased MDSCs might result in an unfavorable cycle leading to inflammatory amplification, allowing the disorder to progress towards AD with inflammation. The 3-month-old 5xFAD mice used in the present study mimicked mild-to-moderate AD in human beings, so the proportion and suppressive function of MDSCs might start to decline. Here comes the question: Pg can increase the proportion and function of mMDSCs in healthy conditions while not quite clear in mild-to-moderate AD. In our study, Pg infection increased the proportion of MDSCs in peripheral blood and spleen of 5xFAD mice, where mMDSCs were significantly decreased in the proportion and immunosuppressive function after Pg infection, while gMDSCs were found no change. Furthermore, exogenous supplement of mMDSCs increased endogenous mMDSCs especially in bone marrow and decreased gMDSCs especially in blood.

Lipopolysaccharide, gingipain, fimbria and peptidyl arginine deiminase are the key virulence factors of Pg [26, 35, 36]. These virulence factors facilitate the inflammatory induction and evasion of immune surveillance. It is a limitation of our study that we did not figure out which key virulence factor of Pg accounted for the change of proportion and immunosuppressive function of mMDSCs. There is a report that fimbria of Pg can activate ICAM-3 of dendritic cells, which helps Pg survive in the body for a long time, resulting in the body in a state of chronic inflammation [37]. Perhaps, the fimbria of Pg may decrease the expression of Arg1 and Nos2 and then influence the proportion and immunosuppressive function of mMDSCs.

### Contribution of mMDSCs in rescuing immune homeostasis impaired by Pg in 5xFAD mice

The primary function of mMDSCs is the suppressive capacity of immune cells, including T and B lymphocytes via L-arginine depletion through Arg1 and Nos2 enzymatic activity. Meanwhile, mMDSCs may enhance the proportion of other immunosuppressive cells, such as Treg and Breg. Treg have diverse subsets of which the best-defined subset constitutively expresses CD25 and FOXP3 [38]. In analogy, Breg have various phenotypes of which the majority are IL-10-competent cells named B10 cells and become the main source of IL-10 expressed by B cells [39, 40]. In the current study, the relative expressions of Arg1 and Nos2 in spleen and bone marrow were decreased after Pg infection and increased after mMDSCs injection. In addition, the proportion of CD4^+^ IFN-γ^+^ T cells, namely, T helper 1 (Th1) cells in MLN, increased after Pg infection while decreased after mMDSCs injection. Therefore, it is possible that Pg can down-regulate Arg1 and Nos2 expression and damage the immunosuppressive function of mMDSCs, while exogenous mMDSCs can rescue the suppressive function of endogenous mMDSCs via up-regulation of Arg1 and Nos2 expression. It is also one of the limitations in this study that we only analyzed entire spleen and bone-marrow cells but not purified mMDSCs to examine the relative mRNA expressions. In addition, the mRNA level is not necessarily in line with enzyme activities, so further studies are pivotal to clarify the functional differences in Arg1 and Nos2 of mMDSCs in vivo. Besides, though there were no significant differences in the proportions of Treg among the three groups, the proportion of B10 cells in spleen decreased significantly after Pg infection while tended to increase after mMDSCs injection. It is consistent with our previous study that Pg infection could downregulate B10 cells [7]. Moreover, the proportions of IL-10^+^ mMDSCs in bone marrow decreased after Pg infection while that in blood increased after mMDSCs injection. In addition, the proportions of IL-10^+^ gMDSCs in spleen decreased after Pg infection while increased after mMDSCs injection. Taken together, Pg could damage the immunosuppressive function of mMDSCs by those three ways, and exogenous supplement of mMDSCs could reverse the three ways to improve the suppressive function of endogenous mMDSCs. In addition, our results showed that the proportions of CD4^+^ IL-6^+^ and CD8^+^ IL-6^+^ T cells in MLN increased after Pg infection while decreased after mMDSCs injection. The proportion of CD8^+^ IL-6^+^ T cells in blood also increased after Pg infection while decreased after mMDSCs injection. The proportion of CD4^+^ IL-10^+^ T cells in MLN and spleen decreased after Pg infection while increased after mMDSCs injection. The reduced IL-6-producing cells and raised IL-10-producing cells might lead to a more anti-inflammatory immune microenvironment in AD after exogenous mMDSCs injection.

Neuroinflammation is existent throughout the course of AD. Among the innate immune cells in the central nervous system (CNS), microglia are the primary participants in neuroinflammation [41]. Microglia possess similar functions to peripheral myeloid cells including chemotaxis, phagocytosis, and cytokine expression. Recent studies have demonstrated broad neuro-immune cross-talk between the central and peripheral immune system throughout the whole pathogenesis of AD. The cross-talk may stem directly or indirectly from peripheral immune cells due to higher permeability of blood–brain barrier in neurodegenerative diseases. Peripheral myeloid cells can move into the CNS and function during inflammatory insult and microglial depletion. It is reported that MDSCs can infiltrate the cerebral lesion site and suppress the activation of pro-inflammatory microglia in the CNS in traumatic brain injury [42]. Hence, MDSCs might have an influence on the development and outcomes of AD via potent immunosuppressive function on microglia. Indeed, microglia play a dual role in the CNS. On the one hand, microglia can fight against different bacterial antigens and eliminate waste products aggregated in the brain. On the other hand, microglia can produce pro-inflammatory cytokines, such as IL-6 and IL-1β, which exacerbates neuroinflammation in AD. Based on morphology and immunochemical markers, microglia are defined as two subpopulations with pro-inflammatory M1 phenotype and anti-inflammatory M2 phenotype. Activation and polarization of resting microglia into M1 phenotype are stimulated by bacterial-derived compounds and cytokines generated by Th1 cells and astrocytes. M1 phenotype secretes proteolytic enzyme, redox signaling molecules, and pro-inflammatory cytokines. In contrast, microglia are polarized towards M2 phenotype through the stimulation of cytokines produced by Th2 cells. M2 phenotype produces scavenger receptors and anti-inflammatory cytokines, such as IL-10. Three subclasses of M2 phenotype function in inflammation suppression (M2a) and tissue regeneration (M2c), whereas M2b still remains unknown [43]. In the current study, Pg infection down-regulated mMDSCs in AD, and increased the proportions of total microglia while decreased the M2 phenotype, suggesting a pro-inflammatory state of the brain. Furthermore, exogenous supplement of mMDSCs up-regulated endogenous mMDSCs, and increased the proportions of total microglia and the M2 phenotype, indicating an anti-inflammatory condition in the CNS and protecting the cerebral neurons.

As mentioned above, exogenous mMDSCs injection may reduce the cognitive impairment, neuronal loss and Aβ deposition exacerbated by Pg infection through rescuing immune homeostasis.

### Prospective of AD therapy based on MDSCs modulation

Promising potential can be seen of cell-based therapies to treat various CNS disorders. In this context, bone-marrow cells are one of the most suitable and attractive sources, since they can be easily acquired and induced with rapid proliferation afterward, and could be used for autologous as well as allogeneic transplantation through intravenous or intrathecal injection [44, 45]. In the field of traumatic spinal cord injury, transplantation of mMDSCs at lesion areas significantly attenuates acute inflammation and promotes tissue repair, thereby improving the neurological outcomes [46]. Even administration of GM-CSF alone shows neuroprotective effects and improved functional recovery [47]. The present study exhibited that Pg promoted AD through impairing mMDSCs in proportion and immunosuppressive function, exacerbating immune-overreaction. Moreover, supplement of exogenous mMDSCs could inhibit immune-overreaction and ameliorate Pg-promoted neuroinflammation as well as cognitive impairment. With the increasing incidence and prevalence of AD and periodontitis, it is of great value to explore therapies based on MDSCs modulation. One limitation of this study was not tracking the injected mMDSCs in 5xFAD mice. Further studies may be required to clarify the fate of cells in vivo and determine the most effective method.

## Conclusions

Oral Pg infection can exacerbate neuroinflammation and cognitive impairment of AD through down-regulation the proportion of mMDSCs in vivo. Moreover, exogenous supplement of mMDSCs can up-regulate the proportion of mMDSCs, recover immune homeostasis, ameliorate neuroinflammation and cognitive impairment of AD exacerbated by Pg infection.

## Supplementary Information

**Additional file 1: Fig. S1.** Schematic diagram of partial results of mouse genotyping. PCR products with simultaneous 377 bp (APP) and 608 bp (PS1) were identified in 5xFAD mice; Littermates only had 324 bp PCR products. **Fig. S2.** Body weight of 5xFAD mice during experiment. There was no significant difference in body weight of three groups of mice during the whole experiment.

## Data Availability

All data generated or analyzed during this study are included in this article and its additional information files.

## Abbreviations

5xFAD: Five familial Alzheimer’s disease mutations; Aβ: Beta-amyloid; AD: Alzheimer’s disease; APP: Amyloid precursor protein
Arg1: Arginase 1
B10: IL-10-competent regulatory B cells
Breg: Regulatory B cells
CFU: Colony-forming units
CMC: Carboxymethyl cellulose
CNS: Central nervous system
GM-CSF: Granulocyte–macrophage colony stimulating factor
gMDSCs: Granulocytic myeloid-derived suppressor cells
Iba-1: Allograft inflammatory factor 1
IFN-γ: Interferon γ
IL-1β: Interleukin 1β
IL-6: Interleukin 6
IL-10: Interleukin 10
LPS: Lipopolysaccharide
MDSCs: Myeloid-derived suppressor cells
MLN: Mesenteric lymph nodes
mMDSCs: Monocytic myeloid-derived suppressor cells
MWM: Morris water maze
Nos2: Inducible nitric oxide synthase
OFT: Open field test
Pg: *Porphyromonas gingivalis*
PMA: Phorbol 12-myristate 13-acetate
PS1: Presenilin 1
PS2: Presenilin 2
Th1: T helper 1 cells
TQ: Target quadrant
Treg: Regulatory T cells
