# Supplementary material for: Exogenous monocyte myeloid-derived suppressor cells ameliorate immune imbalance, neuroinflammation and cognitive impairment in 5xFAD mice infected with Porphyromonas gingivalis
Source: J Neuroinflammation. 2023 Mar 2;20:55. doi: 10.1186/s12974-023-02743-8 (PMC9979576; doi:10.1186/s12974-023-02743-8)
Supplement: Supplementary file 1 — Additional file 1: Fig. S1. Schematic diagram of partial results of mouse genotyping. PCR products with simultaneous 377 bp (APP) and 608 bp (PS1) were identified in 5xFAD mice; Littermates only had 324 bp PCR products. Fig. S2. Body weight of 5xFAD mice during experiment. There was no significant difference in body weight of three groups of mice during the whole experiment. [file 12974_2023_2743_MOESM1_ESM.docx]

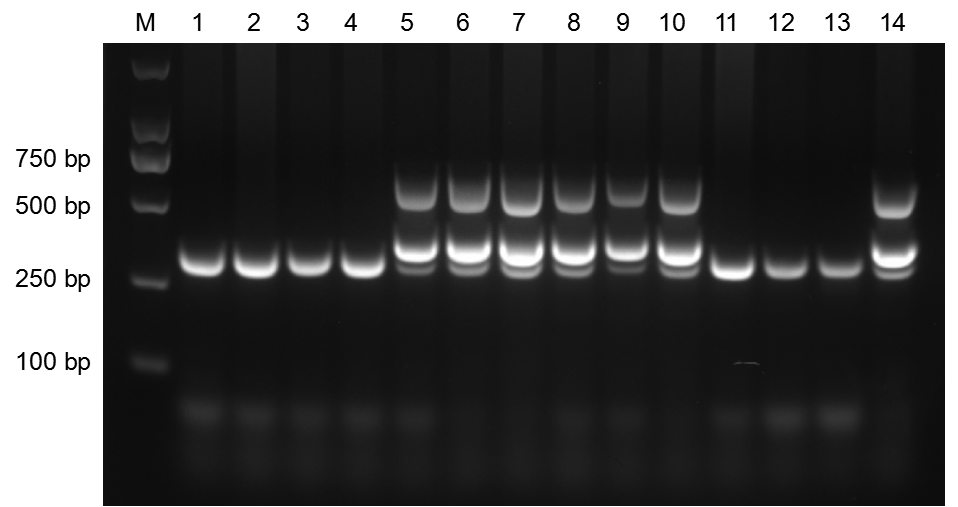


**Fig. S1** Schematic diagram of partial results of mouse genotyping. PCR products with simultaneous 377 bp (APP) and 608 bp (PS1) were identified in 5xFAD mice; Littermates only had 324 bp PCR products.


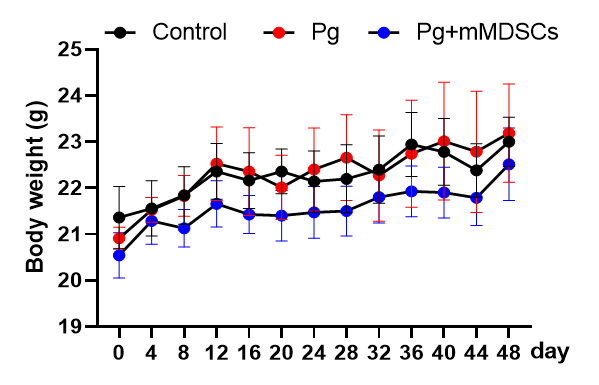

**Fig. S2** Body weight of 5xFAD mice during experiment. There was no significant difference in body weight of three groups of mice during the whole experiment.

.
